# Ex vivo comparison of ACU193 and lecanemab reveals binding differences in mouse brain

**DOI:** 10.1002/alz.71509

**Published:** 2026-06-01

**Authors:** Martine B. Grenon, Erika N. Cline, Jasna Jerecic, Elizabeth Johnson, Eric Siemers, Cynthia A. Lemere

**Affiliations:** ^1^ Building for Transformative Medicine Brigham and Women's Hospital Boston Massachusetts USA; ^2^ Department of Neurology Harvard Medical School Boston Massachusetts USA; ^3^ Faculty of Psychology and Neuroscience Maastricht University Maastricht the Netherlands; ^4^ Acumen Pharmaceuticals Newton Massachusetts USA

**Keywords:** amyloid‐related‐imaging abnormalities, anti‐Aβ monoclonal antibody, cerebral amyloid angiopathy, immunofluorescent staining

## Abstract

**INTRODUCTION:**

Immunotherapy targeting amyloid beta (Aβ) is limited by amyloid‐related imaging abnormalities (ARIA), hypothesized to result from the direct binding of anti‐Aβ monoclonal antibodies (mAbs) to vascular cerebral amyloid angiopathy (CAA), eliciting an immune response.

**METHODS:**

Immunofluorescent staining was used to characterize ex vivo plaque and vascular binding of recombinant FDA‐approved lecanemab and clinical candidate sabirnetug (ACU193) in 30 min and 24 h fixed APP:hE4 mouse brain tissue.

**RESULTS:**

Fixation time was a key factor influencing Aβ antigen labeling with prolonged fixation differentially affecting pan‐Aβ and mAb immunoreactivity. Compared with ACU193, lecanemab showed greater cortical plaque and cerebellar vascular labeling and encompassed a larger fraction of total pan‐Aβ and β‐pleated‐sheet‐rich signal, reflecting both mAb target abundance and selectivity.

**DISCUSSION:**

These findings emphasize that experimental protocols and antibody properties jointly shape the observed mAb binding patterns and highlight differences that may contribute to antibody‐specific ARIA risk observed clinically.

## Highlights

- This study compared immunostaining of two clinical anti‐Aβ antibodies in brains of APP:hE4 mice.
- Serial sections were double labeled with pan‐Aβ antibody or β‐sheet dye across concentration series.
- Fixation time and mAb concentration strongly influenced Aβ antigen labeling patterns.
- Lecanemab labeled more cortical plaques and cerebellar vascular Aβ than ACU193.

## 1 BACKGROUND

Alzheimer's disease (AD) is a progressive neurodegenerative disease characterized by the gradual accumulation of amyloid beta (Aβ) plaques and neurofibrillary tau tangles in the brain. Aβ is an ∼4.5‐kilodalton peptide containing 39 to 42 amino acid residues.^1^, ^2^ Aβ monomers are flexible, partially hydrophobic, and can misfold and self‐associate into many conformations with differing propensities for aggregation.^3^, ^4^ A variety of morphologies are known to co‐exist along the aggregation pathway, ranging from low‐molecular‐weight oligomers to high‐molecular‐weight oligomers to protofibrils to insoluble fibrils, which accumulate to form extracellular Aβ plaques.^5^ Early research focused on insoluble aggregates, but attention has shifted toward soluble Aβ species, particularly oligomers and protofibrils, as driving synaptic dysfunction and neuronal loss.^3^, ^6^, ^7^, ^8^, ^9^, ^10^ Studies have suggested that Aβ oligomers correlate with disease severity,^11^, ^12^ and in amyloid mouse models cognitive deficits can appear before plaque deposition or the detection of fibrils.^13^, ^14^

Anti‐Aβ immunotherapy is the first disease‐modifying treatment for AD, and at least eight clinical antibodies have been developed targeting diverse forms of Aβ.^15^ In the last 4 years, three anti‐Aβ monoclonal antibodies (mAbs) have received US Food and Drug Administration (FDA) approval for the treatment of early‐stage AD: Biogen's Aduhelm (aducanumab, approved 2021, later discontinued),^16^ Eisai/Biogen's Leqembi (lecanemab, approved 2023),^17^ and Eli Lilly's Kisunla (donanemab, approved 2024).^18^ After magnetic resonance imaging (MRI) abnormalities were first observed with bapineuzumab, a workgroup coined the term “amyloid‐related imaging abnormalities (ARIA),” encompassing ARIA‐edema/effusions (ARIA‐E), MRI signal alterations thought to represent vasogenic edema and related extravasated fluid phenomena, and ARIA‐hemosiderin deposition (ARIA‐H), MRI signal alterations attributable to microhemorrhage and hemosiderosis.^19^ A 2025 meta‐analysis of phase 3 clinical trials of anti‐Aβ immunotherapies reported an overall ARIA‐E incidence of 33.7% and ARIA‐H incidence of 17.8%, with significantly higher rates in apolipoprotein E (*APOE*) ε4 carriers.^20^ ARIA‐E events during clinical trials have been predominantly asymptomatic, resolving within 3 to 4 months with dose adjustment, suspension, or discontinuation.^21^ ARIA‐H events are more permanent. When symptomatic, ARIA‐E is usually mild to moderate, with infrequent severe cases (1% to 2%) requiring hospitalization and intensive management.^21^ Macrohemorrhages are rare and usually asymptomatic and generally lead to treatment discontinuation.^21^ However, several fatal cases have been reported, including, for example, a patient receiving lecanemab who developed multiple cerebral hemorrhages after thrombolysis for acute ischemic stroke.^22^

**RESEARCH IN CONTEXT**

1. **Systematic review**: We searched PubMed for studies examining tissue fixation effects on antigenicity, the characterization of cerebral amyloid angiopathy (CAA) and plaque pathology in AD and AD mouse models, and the development and evaluation of clinical anti‐Aβ antibodies, including their epitopes, selectivity, and pre‐clinical and clinical data, and reported ARIA incidence.
2. **Interpretation**: This study provides the first ex vivo, head‐to‐head quantitative immunohistochemical comparison of clinical anti‐Aβ antibodies. Our results show that fixation time and concentration affected immunoreactivity and demand optimization in future studies of similar scope. Lecanemab exhibited greater plaque and vascular labeling than ACU193, reflecting differences in target specificity and/or abundance. Differences in binding among antibodies may help interpret future variability in ARIA incidence as additional clinical data emerge.
3. **Future directions**: Future work should extend these analyses to human AD tissue to validate the observed staining profiles. Expanding comparisons to include additional clinical anti‐Aβ antibodies, and relating vascular binding patterns to clinical ARIA outcomes will be essential to clarify how vascular immunoreactivity relates to ARIA risk.

The exact mechanisms of ARIA‐E/H remain unknown and several hypotheses have been presented.^23^ Circumstantial evidence suggests that ARIA‐E is initiated by the direct binding of anti‐Aβ mAbs to Aβ accumulated in cerebral blood vessel walls, cerebral amyloid angiopathy (CAA).^24^, ^25^, ^26^, ^27^ This hypothesis is supported by evidence of ARIA following anti‐Aβ immunotherapy in AD mouse models with CAA, which show a propensity to develop ARIA‐H with chronic anti‐Aβ mAb treatment,^24^, ^27^, ^28^, ^29^, ^30^ and in AD patients with CAA, which increases the risk of ARIA.^31^, ^32^ *APOE* ε4 is associated with both CAA incidence and ARIA‐E/H risk.^33^, ^34^ Additionally, CAA‐related inflammation, a rare autoimmune encephalopathy triggered by anti‐Aβ autoantibodies, which leads to vasogenic edema and hemorrhage, shares radiographic findings similar to those of ARIA‐E/H.^31^, ^35^, ^36^

Absolute ARIA rates vary between clinical mAbs,^20^ which may reflect differences in their Aβ binding profiles. Clinical anti‐Aβ mAbs target distinct epitopes along the Aβ peptide, which may appear on more than one assembly state. As a result, antibodies described as monomer‐preferring may still bind to larger aggregates, while fibril‐preferring mAbs may also bind smaller soluble species.^3^ CAA is mostly composed of insoluble Aβ species,^37^, ^38^ which may influence antibody binding. Thus, Aβ antibodies that bind strongly to CAA may lead to a higher incidence of ARIA. We hypothesize that comparing cerebrovascular Aβ binding among mAbs may be a first step in evaluating their relative risks for ARIA. In addition to binding characteristics, other factors, such as titration scheme and degree of effector functioning, may influence the risk of ARIA. Here, we report an ex vivo side‐by‐side immune‐labeling comparison of two human clinical mAbs, Acumen's sabirnetug (ACU193; IgG2) and recombinant lecanemab (IgG1), based on Eisai/Biogen's Leqembi, for plaque versus vascular Aβ in APP:hE4 mice.

## 2 METHODS

### 2.1 Animals

This study utilized male and female *APP^NL‐F/NL‐F^:hAPOE4* ^*fl/fl*^ (APP:hE4) mice. APP:hE4 mice were originally generated by crossing *APP^NL‐F/NL‐F^* mice on a C57BL/6J background^39^, ^40^ with human targeted replacement *APOE^ε4/ε4^* floxed mice on a C57BL/6NTac background,^41^ as previously described.^42^ Dr. Takaomi Saido (RIKEN Institute) provided a material transfer agreement (MTA) permitting the crossing of the *APP^NL‐F/NL‐F^* line with the targeted replacement human *APOE* mice, and the Cure Alzheimer's Fund provided a MTA for the *APOE^ε4/ε4^* floxed line. Breeding pairs of APP:hE4 mice were provided by Dr. David Holtzman (Washington University School of Medicine). Animals were 19 to 21 months of age at the time of sacrifice, and both sexes were included (*n* = 6; 2 F, 4 M). Details are provided in the Supplementary Material (Table S1). The colony was maintained in a barrier facility at a constant temperature with a 12:12 light/dark cycle and housed based on sex. Water and food (PicoLab® Rodent Diet 20 5053) were available ad libitum. All animal studies were approved by the Institutional Animal Care and Use Committees at Brigham and Women's Hospital (Protocol Number: 2016N000396).

### 2.2 Euthanasia and tissue preparation

**FIGURE 1:**
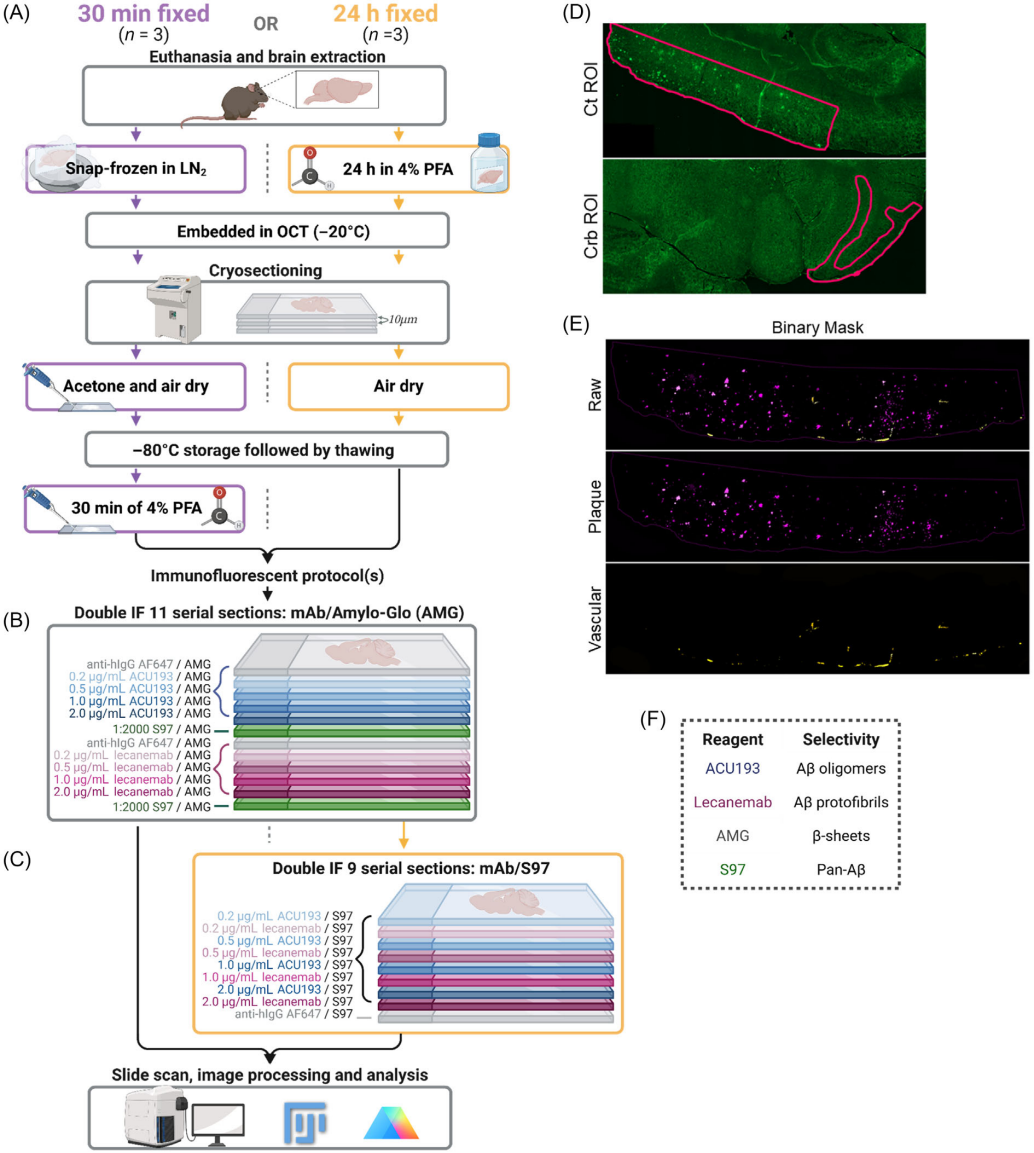
Methods. (A) Six 20‐month‐old APP:hE4 mice were split into 30 min and 24 h *post mortem* fixation groups (*n* = 3 each). Brains were bisected. The 30 min group was snap‑frozen, embedded in OCT, maintained at −20°C, sectioned, and later post‑fixed in 4% PFA for 30 min; the 24 h group was fixed in 4% PFA for 24 h, transferred to graduated sucrose cryo‐protection solutions, embedded in OCT, maintained at −20°C, and sectioned. (B) In both fixation groups (*n* = 6), 11 serial sections per mouse were double‐labeled with a mAb (ACU193 or lecanemab) and AMG. Each mAb was tested at 0.2, 0.5, 1.0, and 2.0 µg/mL. Sections stained with S97 (1:2000) served as positive controls, and sections processed without a primary antibody served as negative controls. The series was repeated for the second mAb. (C) In the 24 h fixation group only (*n* = 3), nine serial sections per mouse were double‐labeled with an anti‐Aβ mAb (ACU193 or lecanemab) and S97 (1:2000). ACU193 and lecanemab were alternated between adjacent sections, each tested at 0.2, 0.5, 1.0, and 2.0 µg/mL. One section processed without a mAb primary antibody served as a negative control. (D) For image processing, one region of interest (ROI) was selected in the cortex and one in the cerebellum, chosen based on high pathology presence, and subsequently analyzed. (E) Representative images illustrating the image‐processing workflow. Staining was evaluated using two metrics: (i) vascular and (ii) plaque. The top panel shows a thresholded fluorescent channel with plaque and vascular signals displayed in different colors for illustration. The middle and bottom panels show the corresponding binary masks used for analysis. For quantification, each channel was thresholded to generate binary masks and off‑target signal was manually erased prior to calculation of percent area immunoreactivity and channel colocalization. (F) Table summarizing primary reagents used and their reported selectivity. mAb, monoclonal antibody (ACU193, lecanemab); AMG, amylo‐glo; Crb, cerebellum; Ct, cortex; LN_2_, liquid nitrogen; OCT, optimal cutting temperature; PFA, paraformaldehyde; ROI, region of interest. Created in BioRender. Grenon, M. (2026) https://BioRender.com/7ah9em5.

Mice were euthanized by excess carbon dioxide inhalation, exsanguinated, and subjected to bilateral thoracotomy, followed by transcardial perfusion with phosphate buffered saline (PBS; Invitrogen, AM9625). Brains were then removed and divided sagittally. One hemibrain per mouse was selected for use in this study. Mice were assigned to a fixation group, as illustrated in Figure 1A: 30 min (*n* = 3; 1 F, 2 M) or 24 h (*n* = 3; 1 F, 2 M). Hemibrains in the 30 min group were snap‐frozen in liquid nitrogen. Hemibrains in the 24 h group were immersion fixed in 4% paraformaldehyde (PFA; Electron Microscopy Sciences, 15714S) for 24 h, then cryoprotected with 10% and 30% sucrose solutions. For cryosectioning, hemibrains were embedded in Optimal Cutting Temperature (OCT) compound (Fisher Healthcare Tissue‐Plus, 23‐730‐571), frozen, and sectioned at 10 µm using a cryostat (Epredia CryoStar NX70) maintained at −20°C. Sections were mounted directly onto glass slides (Fisherbrand Superfrost Plus, 1255015; Fisher Colorfrost Plus, 1255017). For the 30 min group, slide‐mounted sections were briefly exposed to 100% acetone (Fisher Chemical, A18‐1). All sections were then stored at −80°C until use. For immunofluorescence staining, sections were thawed for 90 min prior to protocol initiation. For the 30 min group only, slide‐mounted sections were exposed to 4% PFA for 30 min to improve tissue adherence and staining quality, followed by a PBS wash.

### 2.3 Immunofluorescent staining

Three immunofluorescent staining protocols were performed. A verification staining was first carried out to confirm that cerebrovascular labeling could be identified without an additional vascular marker (Section 2.4). This was followed by two complementary double‐label approaches that comprise the main dataset: (1) human monoclonal antibodies (mAbs; ACU193 and lecanemab) with a styrylbenzene fluorescent histochemical tracer that binds β‐sheet‐rich amyloid aggregates, Amylo‐Glo (AMG; section 2.5)^43^ and (2) mAbs (ACU193 and lecanemab) with a pan‐selective Aβ polyclonal antibody (pAb) S97 that binds fibrillar and non‐fibrillar Aβ (section 2.6).

Unless otherwise specified, all sections were washed three times with distilled water (DS; 5 min), followed by three washes in PBS (5 min). Blocking was performed with 10% normal goat serum (NGS) or normal donkey serum (NDS) in PBS for 1 h at room temperature (RT). The following primary antibodies were diluted in 5% NGS or NDS in PBS and incubated overnight at 4°C: 0.2 to 2.0 µg/mL human anti‐Aβ IgG2 mAb ACU193 (Acumen Pharmaceuticals, Inc., Newton, MA, USA), 0.2 to 2.0 µg/mL recombinant human anti‐Aβ IgG1 mAb lecanemab (Evitria AG, Zurich, Switzerland), 1:2000 rabbit anti‐human Aβ IgG pAb S97 (gift from Dr. Dominic Walsh, BWH, Boston, MA, USA), and 1:400 goat anti‐mouse CD31 IgG pAb (R&D Systems, Minneapolis, MN, USA, AF3628), to detect endothelial cells. The next day, sections were washed three times in PBS (5 min) and incubated with the appropriate Alexa Fluor® conjugated secondary antibody, diluted in 5% NGS or NDS in PBS for 90 min at RT: 1:500 goat anti‐human IgG AF647 (SouthernBiotech, Birmingham, AL, USA, 2040‐31), 1:500 donkey anti‐human IgG AF647 (Jackson ImmunoResearch, West Grove, PA, USA, 709‐605‐149), 1:1000 goat anti‐rabbit IgG AF488 (Invitrogen, Waltham, MA, USA, A‐11034), and 1:500 donkey anti‐goat IgG Cy3 (Jackson ImmunoResearch, West Grove, PA, USA, 705‐165‐147). After the final three PBS washes (5 min), sections were cover‐slipped either using ProLong Gold Antifade Reagent with DAPI (Invitrogen, Waltham, MA, USA, P36931) or in‐house polyvinyl alcohol mounting medium with 1,4‐diazabicyclo[2.2.2]octane (DABCO).

### 2.4 Qualitative validation of mAb vascular labeling

Two serial brain sections from a 24 h fixed APP:hE4 mouse (*n* = 1) were triple‐labeled with 2.0 µg/mL ACU193 or lecanemab, AMG (Biosensis, Thebarton, SA, Australia, TR‐300‐AG), and 1:400 goat anti‐mouse CD31 IgG pAb (R&D Systems, Minneapolis, MN, USA, AF3628). A section without an anti‐Aβ mAb primary antibody was included as a negative control. Prior to coverslipping, sections were stained with AMG according to the manufacturer's instructions. This experiment was performed to qualitatively validate the vascular labeling patterns observed with the amyloid tracer and mAb channels and was not used for quantitative colocalization or vessel‐specific analysis across all experimental sections.

### 2.5 Double labeling of anti‐Aβ mAb and amyloid aggregates

Eleven serial sections (Figure 1B) from each mouse (*n* = 6) were double‐labeled with an anti‐Aβ mAb (ACU193 or lecanemab) and AMG. Each mAb was tested at four increasing concentrations: 0.2, 0.5, 1.0, and 2.0 µg/mL. One section, stained with anti‐Aβ pAb S97 (1:2000), was included as a positive control after the mAb series. One section stained without any primary antibody was included as a negative control before the mAb series. The series was then repeated using the second anti‐Aβ mAb. Prior to coverslipping, sections were stained with AMG according to the manufacturer's instructions.

### 2.6 Double‐label mAb and pan‐Aβ pAb (S97)

Nine serial sections from each 24 h fixed mouse (*n* = 3; Figure 1C) were double‐labeled with an anti‐Aβ mAb (ACU193 or lecanemab) and anti‐Aβ S97 pAb (1:2000). ACU193 and lecanemab were alternated between adjacent sections, with each tested at four increasing concentrations: 0.2, 0.5, 1.0, and 2.0 µg/mL. A section without any primary antibody was included as a negative control. A sequential staining protocol was used to avoid cross‐immunoreactivity. On day 1, sections were incubated overnight with the anti‐Aβ mAb primary antibody. On day 2, sections were washed and incubated with the appropriate secondary antibody. After an additional blocking step, sections were incubated overnight with anti‐Aβ S97 pAb. On day 3, sections were washed, incubated with corresponding secondary antibody, and then washed and mounted as described in Section 2.3.

### 2.7 Microscopy and image processing

Images were acquired using a Zeiss Axioscan 7 Microscope Slide Scanner (Oberkochen, Germany). Immunoreactive signals in the cortex and cerebellum were quantified by Fiji ImageJ software (version 1.54), using custom‐made macros. Two regions of interest (ROIs), one in the cortex and one in the cerebellum, were analyzed. A reference ROI was defined from anti‐Aβ pAb S97 staining in areas of high pathology (Figure 1D) and used to maintain consistent size and location across sections within each mouse. Between mice, ROI size and placement varied modestly to account for tissue integrity and pathology distribution. Staining was evaluated using two metrics: (i) vascular and (ii) plaque. Vascular staining was defined conservatively based on morphology and anatomic distribution as a vessel‐associated labeling pattern, encompassing vascular, perivascular, and leptomeningeal deposition, which could not be reliably distinguished at the resolution of these experiments. Plaque staining encompassed all other labeling not associated with the vascular labeling pattern and was not restricted to compact plaques. Plaque pathology in the cerebellum of APP:hE4 mice was minimal and, therefore, not assessed. Each fluorescent channel was manually thresholded twice to generate binary masks corresponding to the two metrics, plaque and vascular. For each mask, signal belonging to the opposite metric was manually erased before calculating the percent area immunoreactivity and colocalization between fluorescent channels (Figure 1E). This conservative masking approach prioritized unambiguous vascular‐associated labeling but may under‐represent small‐diameter vessels or tangentially sectioned vascular amyloid. For double labeling of anti‐Aβ antibodies with AMG, plaque percent area was quantified from the anti‐Aβ antibody channel and vascular percent area from both the anti‐Aβ antibody and AMG channels. For double labeling of the anti‐Aβ mAbs with anti‐Aβ polyclonal S97, plaque percent area was quantified from both the mAb and S97 channels.

### 2.8 Statistical analysis

All values are reported as mean or group means ± SEM. All data were analyzed using the Prism 10.0 statistical software package from GraphPad (San Diego, CA, USA), and outliers (ROUT) were removed. For statistical analyses, the experimental unit was the tissue section, with each section treated as an independent observation. To assess the effect of fixation, mAbs and concentrations were pooled, and pairwise comparisons were made within each marker (anti‐Aβ mAb, AMG, and S97). To compare overall immunoreactivity of ACU193 and lecanemab, fixation groups and concentrations were pooled, and the two anti‐Aβ mAbs were compared statistically. Other marker groups (AMG, S97, control) were plotted but not included in statistical testing, as they served as positive (AMG, S97) and negative (control) references. Pairwise comparisons were made using Student's *t*‐test or non‐parametric tests such as Welch's *t*‐test or Mann–Whitney U. For statistically tested comparisons, effect sizes were calculated as Hedges’ *g* using group means, standard deviations, and sample sizes. Secondary antibody control‐stained tissue and concentration series data values were not statistically analyzed. Statistical comparisons between individual concentration groups were not performed because each concentration group contained only three sections, with one section representing each mouse. *P* values less than 0.05 (*p* < 0.05) were considered significant for all tests (*p < 0.05, **p < 0.01, ***p < 0.001, ****p < 0.0001).

## 3 RESULTS

To compare antibody labeling patterns relevant to plaque versus cerebrovascular amyloid, immunostaining analyses were performed using two operational categories defined in Methods: vascular and plaque labeling. Vascular labeling was defined based on a vessel‐associated labeling pattern, encompassing vascular, perivascular, and leptomeningeal distribution. Plaque labeling included all remaining antibody staining not associated with the vascular labeling pattern and was not restricted to compacted dense‐core plaques. These categories were applied consistently throughout the Results section.

**FIGURE 2:**
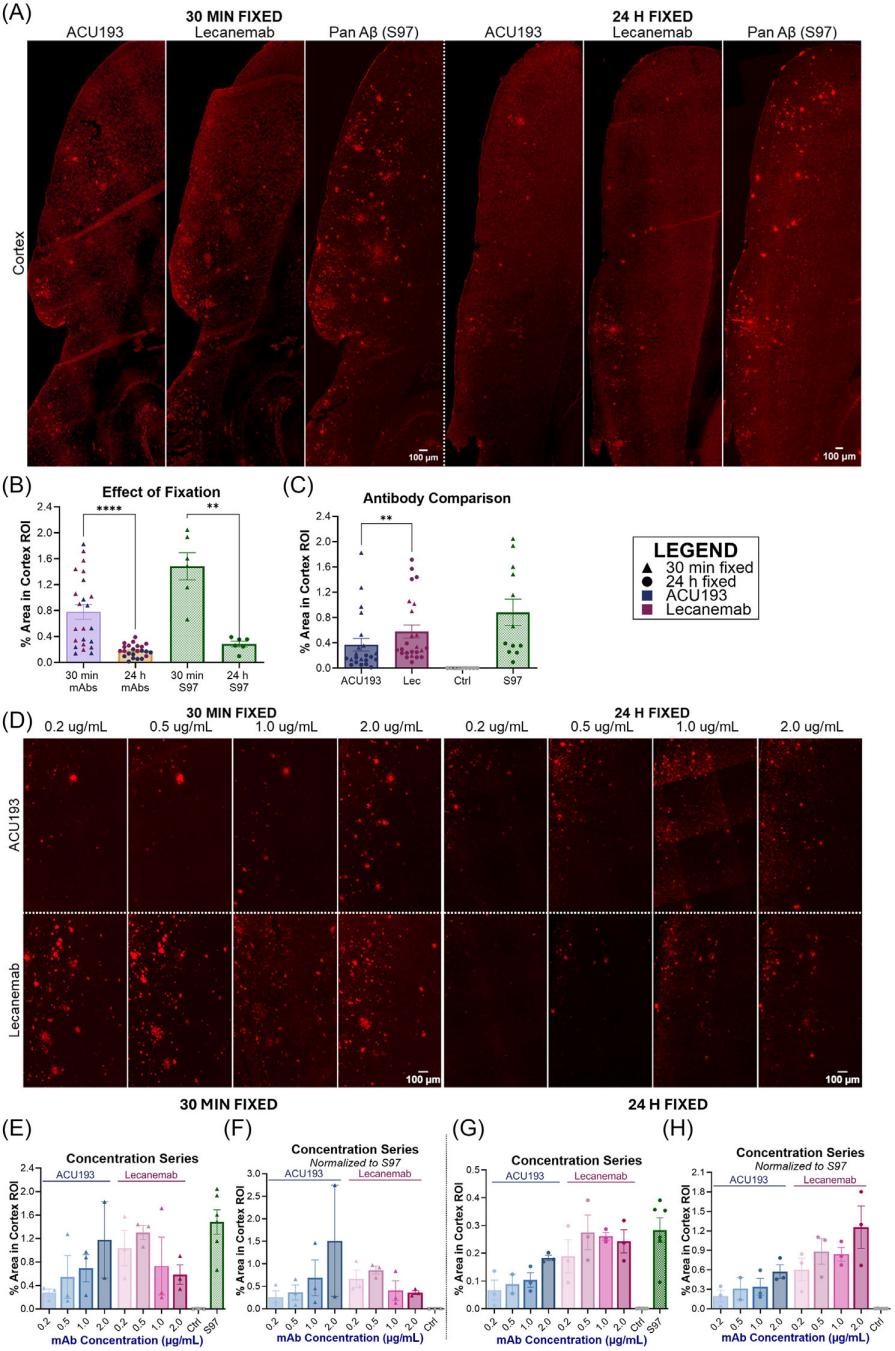
Plaque immunoreactivity in cortex; fixation, antibody, and concentration effects. Serial sagittal sections fixed for 30 min or 24 h were labeled with a mAb (ACU193 or lecanemab; 0.2–2.0 µg/mL). Sections stained with S97 served as a positive control. (A) Representative microphotographs of the cortex from sections stained with 2.0 µg/mL mAb, or 1:2000 S97, across both fixation conditions. (B) Quantification of plaque percent area in cortex ROI by fixation time (pooled across antibodies and concentrations). Fixation effects were assessed separately for mAb and for S97‐labeled plaque area, and corresponding effect sizes are reported as Hedges’ *g* (mAb: *g* = 1.49; S97: *g* = 2.99). (C) Quantification of plaque percent area in cortex ROI by antibody (pooled across fixation times and concentrations). Statistical comparison was restricted to ACU193 versus lecanemab, with effect size reported as Hedges’ *g* (*g *= 0.42); *p < 0.05, **p < 0.01, ***p < 0.001, ****p < 0.0001. (D) Representative microphotographs of cortex of sections stained with 0.2 to 2.0 µg/mL mAb, across both fixation conditions. (E–H) Non‐statistical quantification of percent area in cortex shown separately by fixation condition (not pooled): 30 min fixed tissue (E–F) and 24 h fixed tissue (G–H). Raw values are shown in (E) and (G); values in (F) and (H) are normalized to S97 to account for variability in amyloid load (graphs not statistically analyzed). All values are reported as mean ± SEM. mAb, monoclonal antibody; Ctrl, secondary antibody only control (anti‐human IgG; no primary antibody); Lec, lecanemab.

Plaque labeling by ACU193 and lecanemab was evaluated by immunostaining serial sagittal brain sections of 20‐month‐old APP:hE4 mice and quantifying the percent area of immunoreactivity in a given ROI of the cortex. Plaque pathology was largely confined to the cortex, reflecting the distribution of amyloid deposition in this model, with cerebellar plaques scarce and, therefore, not quantified. Tissue processed using two fixation and tissue preparation protocols, differing primarily in fixation duration (30 min or 24 h), was included to evaluate the effect of prolonged fixation time and associated processing steps on plaque labeling. Each anti‐Aβ mAb was stained at increasing serial concentrations (0.2, 0.5, 1.0, and 2.0 µg/mL). Between each series, S97, a pan‐selective anti‐human Aβ pAb,^44^ was included as a positive control to detect both β‐sheet‐rich and non‐β‐sheet‐rich Aβ deposits (Figure 2A). To assess plaque labeling in the cortex, we first compared the effect of fixation time by combining both mAbs across concentrations. Overall, longer tissue fixation significantly decreased anti‐Aβ mAbs (*p* < 0.0001) and S97 (*p* = 0.0022) labeling (Figure 2B). When data were collapsed across fixation groups, lecanemab showed significantly more labeling than ACU193 (Figure 2C, *p* = 0.0100). S97 labeled more extensively than the anti‐Aβ mAbs but was not included in statistical comparisons, as it served as positive reference (Figure 2C). We next evaluated the effect of mAb concentration (Figure 2D). In 30 min fixed tissue (Figure 2E,F), ACU193 showed a qualitative increase in staining with concentration, while lecanemab labeling appeared consistent across concentrations. In the 24 h fixed tissue (Figure 2G,H), ACU193 again showed a visual increase with concentration. Lecanemab appeared to plateau at 0.5 µg/mL (Figure 2G). However, when normalized to S97 signal, representative of total Aβ burden, a concentration‐dependent pattern emerged (Figure 2H).

**FIGURE 3:**
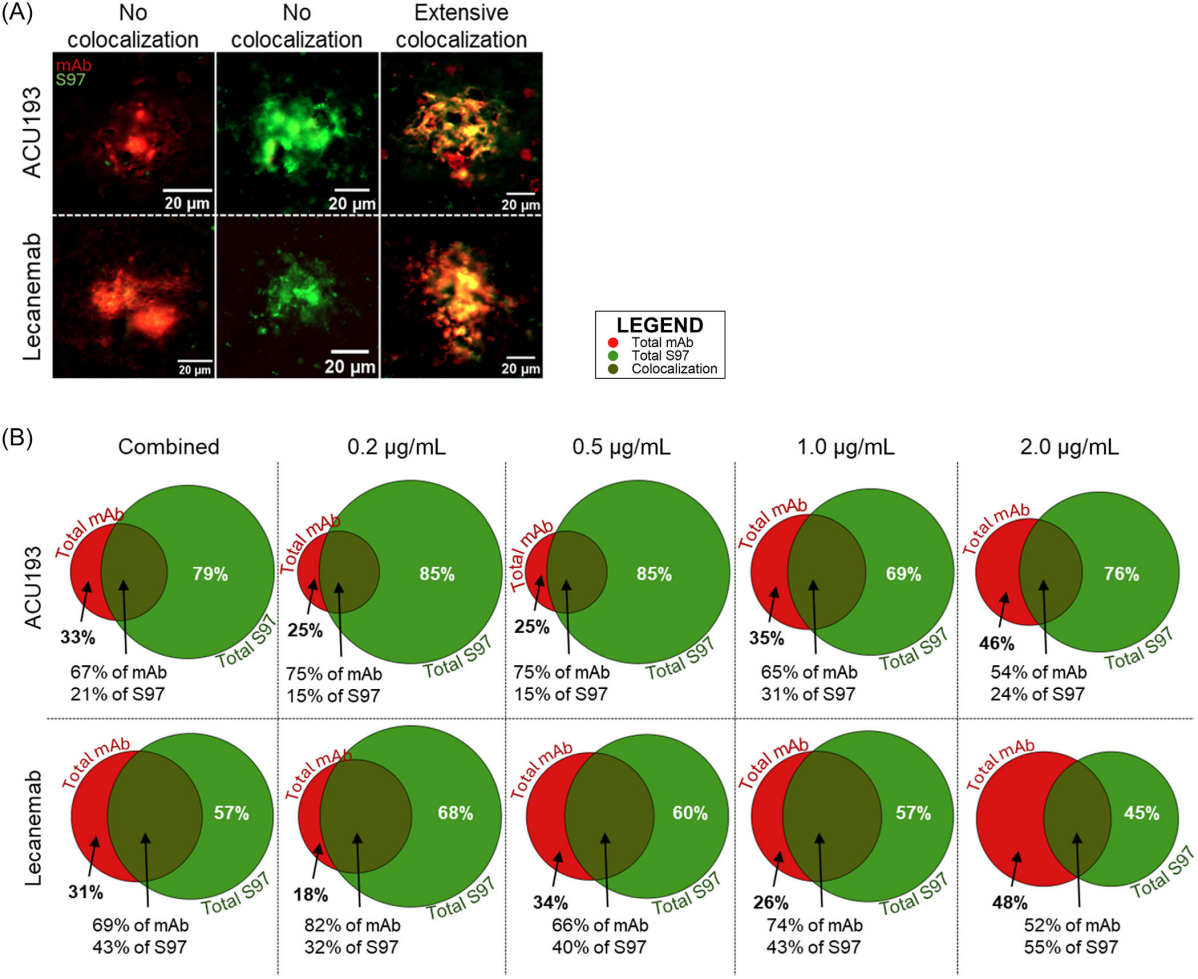
ACU193 and lecanemab cortical plaque staining patterns. (A and B) Serial sagittal sections fixed for 24 h were double labeled with a mAb (ACU193 or lecanemab; 0.2 to 2.0 µg/mL), and S97. (A) Representative merged microphotographs of the cortex from sections stained with 2.0 µg/mL mAb (red) and S97 (green), show examples of mAb staining with no S97 colocalization, S97 staining with no mAb colocalization, and examples of extensive mAb and S97 colocalization. (B) Venn diagrams illustrating overlap between mAb‐positive plaque and S97‐positive plaque staining in cortex. Diagrams depict total mAb‐positive plaque area (red), total S97‐positive plaque area (green), and overlapping fraction (olive green). Percentages represent the mean fraction across mice (expressed as a percentage) of total mAb‐positive plaque area that colocalizes with S97, and independently, the mean fraction of total S97‐positive plaque area that colocalizes with mAb signal. Non‐overlapping regions indicate fraction of mAb‐positive or S97‐positive plaque staining without colocalization. Percentages are normalized within each marker, such that 100% represents the total detected signal for that marker. Circle areas are scaled to preserve the relative overlap of mAb‐positive and S97‐positive staining and do not represent the absolute percent area of mAb‐ or S97‐positive plaque staining within the region of interest. Data are shown for ACU193 and lecanemab with values pooled across concentrations (“combined”) and for each individual antibody concentration. No statistical comparisons were performed. mAb, monoclonal antibody.

To further characterize cortical plaque staining, we double‐labeled serial 24 h fixed sections with either ACU193 or lecanemab together with the pan‐selective Aβ antibody S97. Serial sections were stained with increasing mAb concentrations (0.2, 0.5, 1.0, and 2.0 µg/mL), alternating the mAbs between sections. S97 was used to visualize the global amyloid burden of the section, and colocalization highlighted the subset of signal shared with the mAbs and S97. The percent area of colocalized mAb and S97 plaque labeling was quantified in the cortical ROI. Instances were observed of ACU193 and lecanemab staining without S97 colocalization, as well as S97 staining without mAb colocalization (Figure 3A). Despite differences in overall plaque immunoreactivity, ACU193 and lecanemab each showed a similar proportion of their total signal overlapping with S97, averaging ∼68% when data from all concentrations were combined (Figure 3B). For both antibodies, the fraction of total mAb signal colocalization with S97 declined with increasing mAb concentration, from ∼75% for ACU193 and ∼82% for lecanemab at 0.2 µg/mL to ∼54% and 52% at 2.0 µg/mL, respectively. Conversely, the reciprocal fraction of total S97 signal overlapping with each mAb was consistently greater for lecanemab than for ACU193 across concentrations and when values from all concentrations were combined, with lecanemab overlapping with ∼43% of total S97 signal compared with ∼21% for ACU193. Across concentrations, the fraction of total S97 signal colocalizing with ACU193 varied without a consistent directional pattern, whereas for lecanemab a larger fraction of total S97 signal colocalized at 2.0 µg/mL (∼55%) than 0.2 µg/mL (∼32%; Figure 3B). In addition, we double‐labeled 30 min or 24 h fixed sections with 2.0 µg/mL of either ACU193 or lecanemab together with AMG, which binds β‐sheet‐rich amyloid aggregates and qualitatively compared the plaque staining patterns. As seen in Figure S1, for both mAbs, we observed patterns of staining in the absence of AMG signal, halo‐like staining that surrounded AMG positive cores, and staining that colocalized with AMG‐labeled amyloid aggregates.

To verify that vascular mAb labeling could be reliably identified visually, we performed a triple‐label staining on two serial sections with 2.0 µg/mL ACU193 or lecanemab, AMG, and an endothelial cell marker, anti‐CD31.^45^ Vascular labeling was identified by colocalization with CD31‐positive blood vessels (Figure S2). This experiment validated that vascular staining could be identified by the fluorescent signal in the mAb and AMG channels alone, and subsequent experiments were carried out using double labeling only.

**FIGURE 4:**
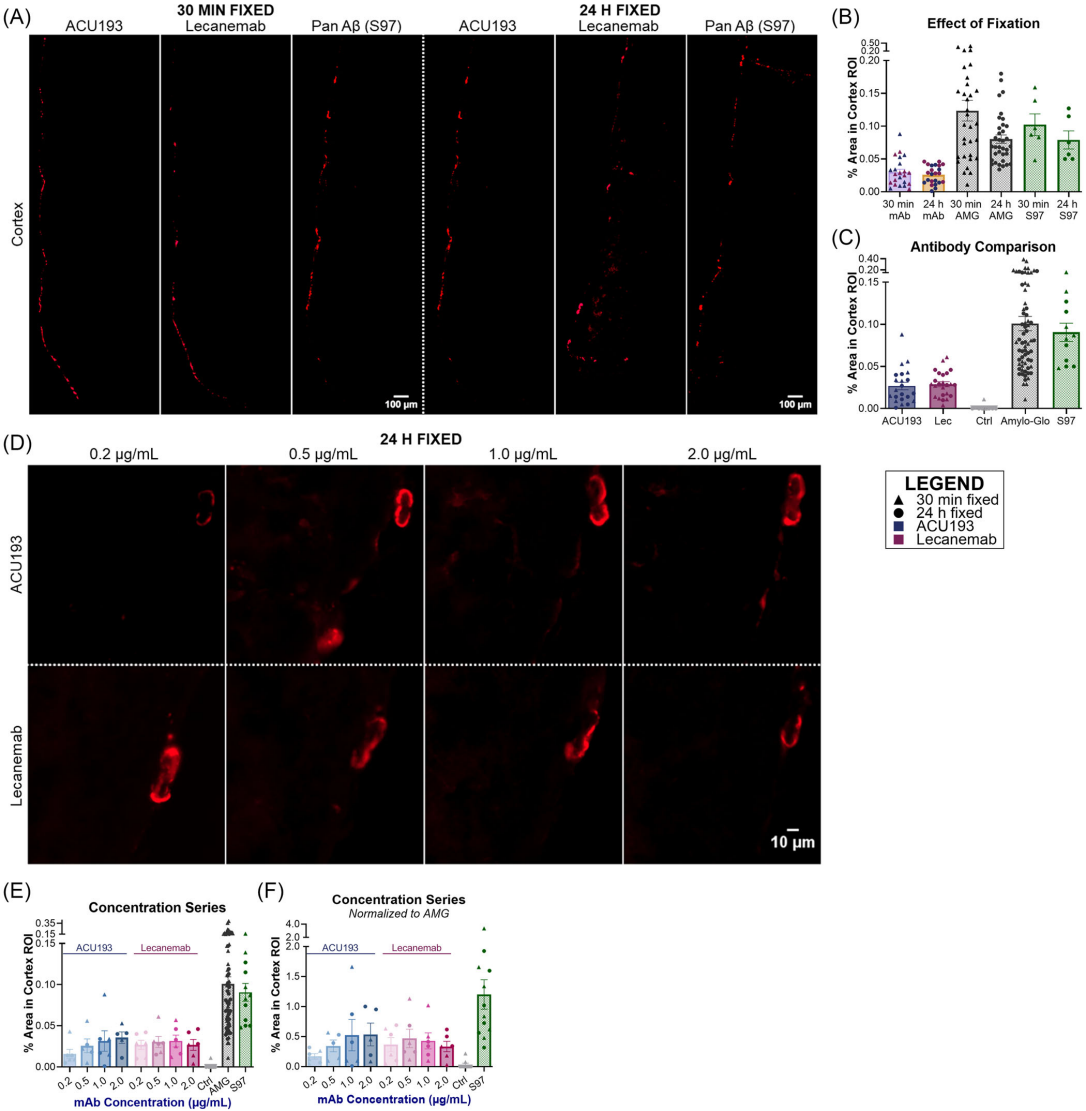
Vascular immunoreactivity in cortex; fixation, antibody, and concentration effects. Serial sagittal sections fixed for 30 min or 24 h were double‐labeled with a mAb (ACU193 or lecanemab; 0.2 to 2.0 µg/mL) and AMG. (A) Representative single‐channel microphotographs of cortex from sections stained with 2.0 µg/mL mAb, or 1:2000 S97, across both fixation conditions. (B) Quantification of vascular percent area in cortex ROI by fixation time (pooled across mAb and concentration). Fixation effects were assessed separately for mAb‐, AMG‐, and S97‐labeled signal, and corresponding effect sizes are reported as Hedges’ *g* (mAb: *g* = 0.21; AMG: *g* = 0.64; S97: *g* = 0.57). (C) Quantification of vascular percent area in cortex ROI by antibody (pooled across fixation and concentration). Statistical comparison was restricted to ACU193 versus lecanemab, with effect size reported as Hedges’ *g* (*g *= 0.11); *p < 0.05, **p < 0.01, ***p < 0.001, ****p < 0.0001. (D) Representative single‐channel microphotographs of leptomeninges of cortex of sections stained with 0.2 to 2.0 µg/mL mAb in 24 h fixation group. (E and F) Non‐statistical quantification of vascular percent area, across both fixation conditions. Raw values are shown in (E); values in (F) are normalized to AMG of the same section to account for variability in amyloid load (graphs not statistically analyzed). All values are reported as mean or group means ± SEM. mAb, monoclonal antibody; AMG, amylo‐glo; Ctrl, secondary antibody only control (anti‐human IgG; no primary antibody); Lec, lecanemab.

Vascular labeling of ACU193 and lecanemab was evaluated by immunostaining serial sagittal brain sections of APP:hE4 mice and quantifying the percentage area of immunoreactivity in the cortex and cerebellum ROIs. Tissue fixed for 30 min or 24 h was included to evaluate the effect of prolonged fixation time (Figure 4A). Serial sections were double‐labeled with ACU193 or lecanemab and AMG. Each mAb was tested at increasing serial concentrations (0.2, 0.5, 1.0, and 2.0 µg/mL). Between each series, S97, a pan‐selective Aβ pAb, was included as a positive control. To assess vascular labeling in the cortex, we first compared the effect of fixation time by combining both mAbs across concentrations. No significant differences were observed between 30 min and 24 h fixed tissue for mAbs (*p* = 0.4762), AMG (*p* = 0.0548) or S97 (*p* = 0.3082; Figure 4B). With data collapsed across fixation groups, ACU193 and lecanemab exhibited similar cortical vascular labeling (Figure 4C, *p *= 0.7003). AMG and the pan‐Aβ antibody showed greater labeling than the mAbs but were not analyzed statistically, as they served as positive references. Given the absence of a fixation effect, we next examined the influence of mAb concentration using data pooled across fixation conditions (Figure 4D). Across concentrations, mean vascular labeling for ACU193 increased qualitatively, from ∼0.016% at 0.2 µg/mL to ∼0.036% area at 2.0 µg/mL, with intermediate values observed at 0.5 µg/mL (∼0.026%) and 1.0 µg/mL (∼0.032% area; Figure 4E). Lecanemab labeling was relatively stable across concentrations, with 0.2 µg/mL (∼0.027% area) displaying staining levels comparable to ACU193 at 2.0 µg/mL (∼0.036% area; Figure 4E). A similar result was observed when mAb signal was normalized to the AMG signal from the same section, serving as a proxy for vascular amyloid burden (Figure 4F). Concentration series are shown in Figure S3, with data plotted separately by fixation protocol.

**FIGURE 5:**
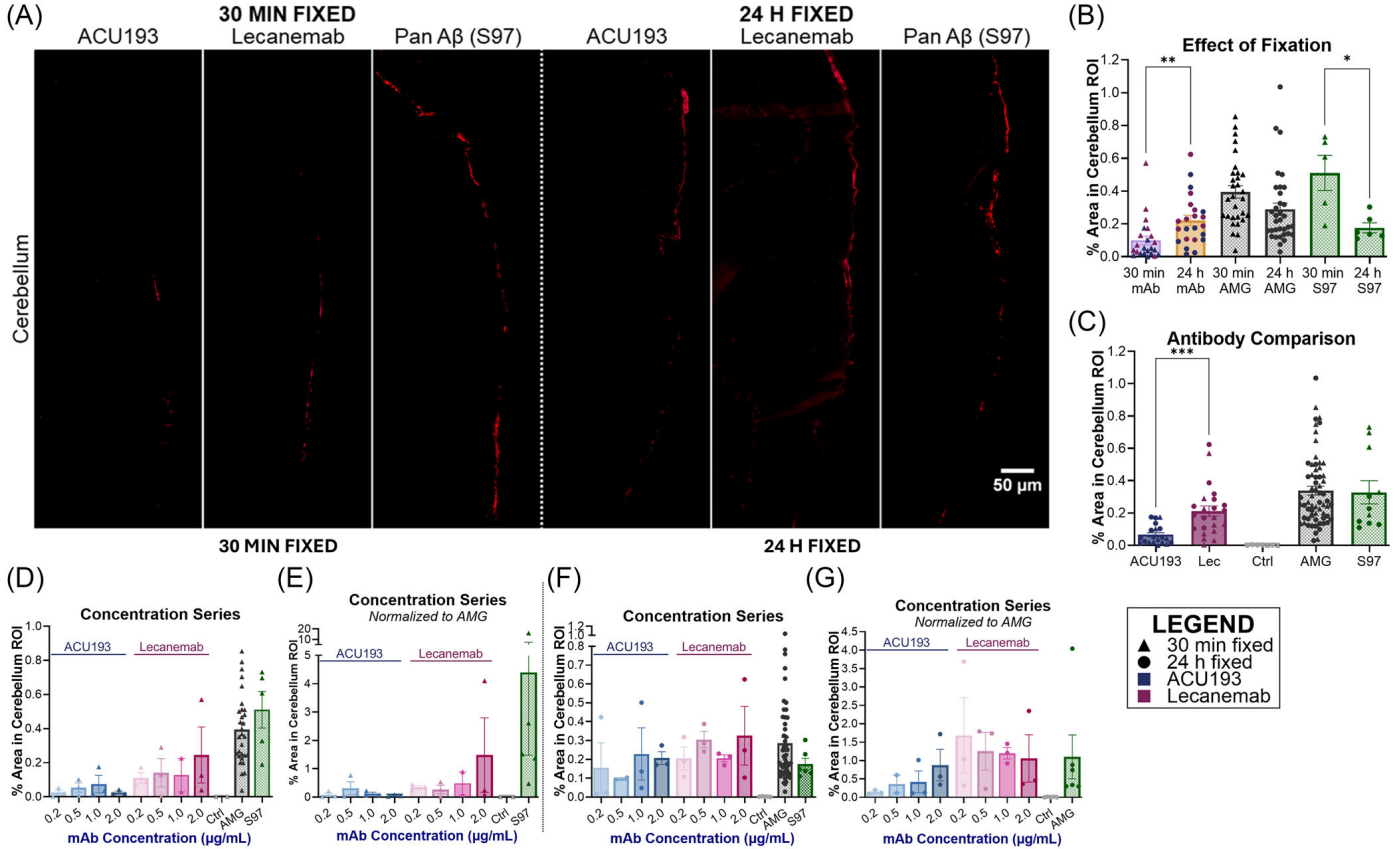
Vascular immunoreactivity in cerebellum; fixation, antibody, and concentration effects. Serial sagittal sections fixed for 30 min or 24 h were double‐labeled with a mAb (ACU193 or lecanemab; 0.2 to 2.0 µg/mL) and AMG. (A) Representative single‐channel microphotographs of cerebellum from sections stained with 2.0 µg/mL mAb, or 1:2000 S97, across both fixation conditions. (B) Quantification of vascular percent area in cerebellum ROI by fixation time (pooled across mAb and concentration). Fixation effects were assessed separately for mAb‐, AMG‐, and S97‐labeled signal, and corresponding effect sizes are reported as Hedges’ *g* (mAb: *g* = 0.85; AMG: *g* = 0.49; S97: *g* = 1.81). (C) Quantification of vascular percent area in cerebellum ROI by antibody (pooled across fixation and concentration). Statistical comparison was restricted to ACU193 versus lecanemab, with effect size reported as Hedges’ *g* (*g *= 1.18); *p < 0.05, **p < 0.01, ***p < 0.001, ****p < 0.0001. (D–G) Non‐statistical quantification of vascular percent area staining in cerebellum in 30 min fixed tissue (D and E) and 24 h fixed tissue (F and G). Raw values are shown in (D) and (F); values in (E) and (G) are normalized to AMG of the same section to account for variability in amyloid load (graphs not statistically analyzed). All values are reported as mean or group means ± SEM. mAb, monoclonal antibody; AMG, amylo‐glo; Ctrl, secondary antibody only control (anti‐human IgG; no primary antibody); Lec, lecanemab.

A parallel analysis of vascular labeling in the cerebellum yielded distinct findings (Figure 5A). When both mAbs were combined across concentrations, the longer fixation time (24 h) increased cerebellar vascular labeling by ACU193 and lecanemab (Figure 5B; *p* = 0.0055). In contrast, the pan‐Aβ antibody S97 showed a significant reduction with longer fixation (*p* = 0.0173), similar to what was observed for plaque labeling in the cortex. No significant differences were observed for AMG (*p* = 0.0509). Independent of fixation effects, when data were combined across fixation groups, lecanemab had significantly more cerebellar vascular labeling than ACU193 (Figure 5C, *p* = 0.0001). Based on pooled mean values, AMG and S97 had the highest levels of labeling, lecanemab was intermediate, and ACU193 the lowest. AMG and S97 were not analyzed statistically, as they served as positive references. Finally, we examined the effect of concentration. In 30 min fixed tissue (Figure 5D,E), ACU193 exhibited minimal staining, while lecanemab appeared to label in a concentration‐dependent manner. In 24 h fixed tissue (Figure 5F,G), normalization to AMG suggested a qualitative, concentration‐dependent increase in ACU193 staining, while lecanemab showed higher percent area across concentrations but with greater variability among mice.

**FIGURE 6:**
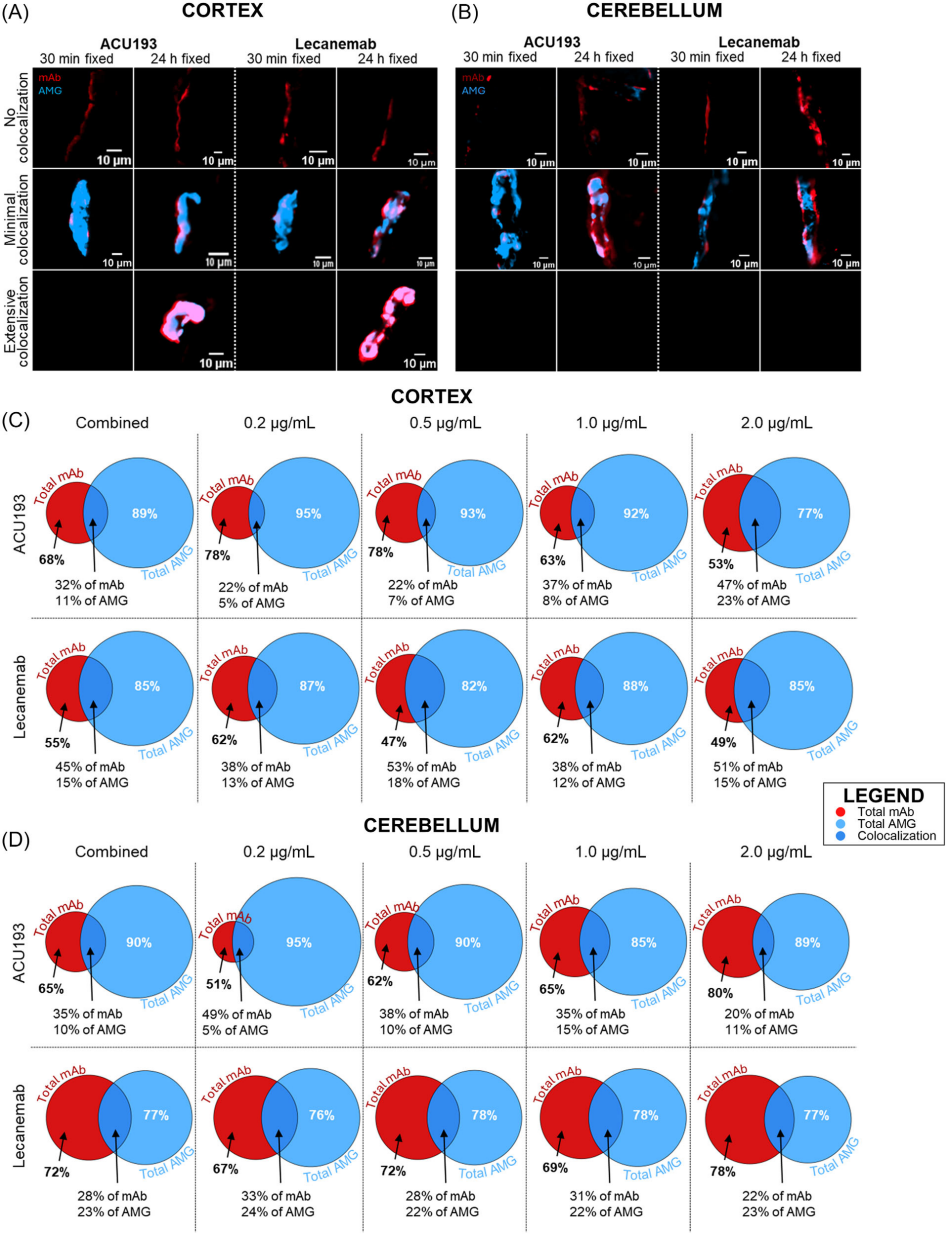
ACU193 and lecanemab vascular staining patterns. Serial sagittal sections fixed for 30 min or 24 h were double‐labeled with a mAb (ACU193 or lecanemab; 0.2 to 2.0 µg/mL) and AMG. Representative merged microphotographs of vascular staining in (A) cortex and (B) cerebellum from sections stained with 2.0 µg/mL mAb (red) and AMG (blue) under both fixation conditions. Qualitative, representative examples are shown illustrating the range of colocalization patterns observed, including vascular labeling with no, minimal, and extensive colocalization of mAb and AMG. (C and D) Venn diagrams illustrating overlap between mAb‐positive vascular staining and AMG‐positive vascular staining in (C) cortex and (D) cerebellum. Diagrams depict total mAb‐positive vascular signal (red), total AMG‐positive vascular signal (light blue), and overlapping fraction (dark blue). Percentages indicate mean fraction across mice (expressed as a percentage) of total mAb‐positive vascular signal that colocalizes with AMG and, independently, the mean fraction of total AMG‐positive vascular signal that colocalizes with mAb. Non‐overlapping regions indicate fractions of total mAb‐positive or AMG‐positive staining without colocalization. Percentages are normalized within each marker, such that 100% represents the total detected signal for that marker. Circle areas are scaled to preserve the relative overlap of mAb‐positive and AMG‐positive staining and do not represent the absolute percent area of mAb‐ or AMG‐positive vascular staining within the region of interest. Data are shown for ACU193 and lecanemab with values pooled across concentrations (“combined”) and for each individual mAb concentration (0.2 to 2.0 µg/mL). No statistical comparisons were performed. mAb, monoclonal antibody; AMG, amylo‐glo.

To further characterize vascular staining, we examined the colocalization of ACU193 or lecanemab with AMG in 30 min and 24 h fixed tissue. The percent area of colocalized mAb and AMG labeling was quantified in the cortical and cerebellar ROIs. Representative images illustrate qualitatively similar staining patterns for ACU193 and lecanemab in both cortex and cerebellum (Figure 6A,B), illustrating the range of colocalization patterns observed. In both regions, examples were observed of mAb labeling with little or no adjacent AMG, as well as AMG‐positive deposits with minimal adjacent colocalized signal or a faint mAb halo. In 24 h fixed cortical and cerebellar tissue (Figure 6B), some deposits showed mAb labeling encircling AMG‐positive regions. In 24 h cortical tissue, instances were observed of a layered pattern with three zones: lateral thin band of mAb labeling, extensive colocalization, and a narrow AMG band medially. Despite similar overall levels of cortical vascular labeling, ACU193 and lecanemab differed in the fraction of total mAb signal overlapping with AMG (Figure 6C). Of the total ACU193 vascular signal, ∼32% colocalized with AMG, whereas ∼45% of total lecanemab signal overlapped with AMG. For ACU193, this fraction increased from ∼22% at 0.2 to ∼47% at 2.0 µg/mL, indicating a concentration‐dependent increase. In contrast, lecanemab fluctuated between ∼38% and ∼53% without a consistent directional trend. In the cerebellum, the relationship was reversed. Despite differences in overall cerebellar vascular labeling, ACU193 and lecanemab showed a similar fraction of total mAb signal overlapping with AMG (Figure 6D). Of the total ACU193 signal, approximately 35% overlapped with AMG, compared with approximately 28% of total lecanemab signal. ACU193 declined from ∼49% at 0.2 µg/mL to ∼20% at 2.0 µg/mL, indicating a concentration‐dependent decrease. Lecanemab, by contrast, fluctuated between ∼28% and ∼33% from 0.2 to 1.0 µg/mL, followed by a reduction to ∼22% at 2.0 µg/mL (Figure 6D). Next, we inverted the analysis to quantify the fraction of total AMG‐positive vascular signal that colocalized with each mAb. In the cortex, when values across concentrations were combined, a greater fraction of total AMG signal colocalized with lecanemab (∼15%) than with ACU193 (∼11%; Figure 6C). This relationship was also observed at the lower antibody concentrations (0.2 to 1.0 µg/mL). In contrast, ACU193 showed a marked increase in AMG colocalization at 2.0 µg/mL, exceeding lecanemab at the highest concentration (Figure 6C). In the cerebellum, the fraction of total AMG signal overlapping with each mAb was consistently greater for lecanemab than for ACU193 across concentrations and when values from all concentrations were combined, with lecanemab overlapping with ∼23% of total AMG signal compared with ∼10% for ACU193 (Figure 6D).

## 4 DISCUSSION

The persistence of cognitive decline after extensive plaque clearance prompted a shift from antibodies recognizing various Aβ conformations to antibodies that selectively target soluble, pathogenic Aβ aggregates.^46^, ^47^ Sabirnetug (ACU193) and lecanemab represent two such clinical antibodies. ACU193, a humanized IgG2 mAb derived from a murine precursor raised against globular Aβ oligomers binding a broad range of soluble aggregates, is currently in a phase 2 clinical trial (NCT06335173).^48^ Lecanemab, a humanized IgG1 mAb derived from a murine precursor raised against Aβ protofibrils, demonstrated clinical efficacy with a relatively low incidence of ARIA‐E (12.6%) and ARIA‐H (17.3%) and is now FDA approved and in clinical use.^17^, ^49^ Prior studies described lecanemab and ACU193 immunostaining in human AD tissue,^48^, ^50^, ^51^ including one report that evaluated both antibodies in parallel but lacked quantitative or vascular‐focused comparisons. Given the observed relationship between CAA and ARIA, we postulate that ARIA risk may be influenced by a mAb's propensity to bind vascular amyloid, together with factors such as IgG isotype and immunogenicity. Here, we compare tissue fixation methods and duration and provide a direct, side‐by‐side quantitative comparison of oligomer‐selective ACU193 and protofibril‐selective lecanemab^52^ on brain sections from APP:hE4 mice.

Immunostaining enables the comparison of plaque and vascular labeling by the anti‐Aβ mAbs while capturing spatial and regional heterogeneity relevant to CAA, but fixation introduces antigenicity loss and epitope masking, requiring protocols to balance structural preservation with labeling efficiency.^53^, ^54^ Twenty‐four‐hour fixation is common in mouse Aβ immunohistochemistry and shorter fixation has been proposed. Here, fixation time strongly influenced Aβ antigen labeling in a region‐specific manner. Plaque detection was generally lower with longer fixation for both mAb and pan‐Aβ antibodies, consistent with reduced detection of soluble Aβ species. For lecanemab, short fixation produced a plateau across concentrations, which may reflect saturation of protofibril binding when soluble Aβ species are well preserved. After longer fixation, when fibrillar Aβ represents a larger proportion of detectable pathology, lecanemab showed a concentration‐dependent increase, consistent with its lower affinity for fibrillar species. Alternatively, prolonged fixation may alter epitope accessibility across concentrations. ACU193 did not exhibit comparable fixation‐dependent shifts. In the cortex, fixation had little effect on vascular labeling by the mAbs, but AMG and pan‐Aβ showed a trend toward reduced labeling with longer fixation, suggesting a modest overall reduction in vascular Aβ detection. In the cerebellum, longer fixation increased mAb labeling while decreasing pan‐Aβ and AMG labeling. This divergence likely reflects regional differences in Aβ conformations between cortex and cerebellum and the distinct Aβ selectivity of the antibodies and AMG, consistent with evidence that aggregate structure strongly influences Aβ mAb binding.^55^ The pan‐Aβ antibody and AMG likely label more compacted fibrillar Aβ species that become less accessible with fixation, while soluble aggregates may remain accessible or stabilized. Reduced immunoreactivity of certain Aβ species after prolonged fixation was reported previously, including pGlu‐3 Aβ.^56^ Thus, direct comparisons between mAb labeling and β‐sheet markers such as AMG should be interpreted with caution, as AMG is a surrogate marker of fibrillar CAA whose signal may be affected differently by fixation. Together, these findings indicate that fixation alters epitope accessibility with effects that depend on both brain region and Aβ species. The balance between morphological preservation and antigenicity is therefore a critical consideration when comparing Aβ antibody binding profiles.

We next examined the plaque binding of the two mAbs. Previous qualitative studies in human AD tissue described lecanemab labeling of both dense‐core and diffuse plaques in the temporal cortex,^50^ ACU193 labeling of diffuse Aβ aggregates with limited colocalization of dense‐core plaque labelling by Thioflavin S in the hippocampus,^48^ and a comparative analysis in the frontal cortex showing plaque staining by both lecanemab and ACU193.^51^ Although fixation protocols were not consistently reported, these patterns are consistent with our qualitative observations. In our quantitative analysis, lecanemab showed significantly greater plaque staining than ACU193. To contextualize these differences, we compared overlap with S97, a pan‐Aβ antibody used as a reference of general Aβ deposition. At lower mAb concentrations, overlap with S97 was high but declined as concentration increased, suggesting that the mAbs labeled Aβ species not detected by S97, likely more diffusely distributed or conformationally distinct soluble Aβ aggregates. Epitope competition may contribute at higher mAb concentrations, where ACU193 or lecanemab may block access to S97. Despite being pan‐selective, S97 may not equally label all Aβ species. S97 showed a greater overlap with lecanemab than ACU193, consistent with the recognition of a broader pool of plaque‐associated Aβ. These differences in plaque staining and pan‐Aβ colocalization likely reflect differences in mAb selectivity and factors such as relative abundance or epitope accessibility of each mAb's target species.

We observed three distinct mAb and AMG colocalization staining patterns, possibly reflecting structural heterogeneity and plaque maturation, with increasing β‐sheet content from diffuse to compact and cored plaques.^57^ (1) Diffuse mAb labeling in the absence of AMG may correspond to early, non‐β‐sheet‐rich Aβ aggregates, including oligomers or protofibrils. (2) Halo‐like mAb staining surrounding AMG‐positive cores is consistent with recognition of soluble Aβ species at the periphery of maturing cored plaques.^58^, ^59^ These staining features were more prominent in 30 min fixed tissue, consistent with the higher plaque mAb signal observed in quantitative analysis. (3) mAb and AMG colocalization may reflect incorporation of soluble Aβ into maturing fibrillar deposits, as biotinylated Aβ oligomers integrate into plaques in vivo and become surrounded by Thioflavin S‐positive amyloid over time.^60^ Together, these patterns align with the differential binding profiles of ACU193 and lecanemab, selective for soluble non‐fibrillar Aβ, and suggest that plaque maturity influences mAb labeling and colocalization with β‐sheet‐rich aggregates. These differences could influence how efficiently each antibody engages low‐abundance soluble versus high‐abundance insoluble Aβ species, a factor previously suggested to impact clinical efficacy by affecting effective dose availability.^47^ Antibodies that mobilize less parenchymal Aβ from neuritic plaques may also be associated with lower ARIA‐E rates.^61^ It is possible that low‐abundance soluble species are saturated but higher mAb concentrations are required to achieve plaque binding, consistent with the view that increased mAb exposure promotes plaque reduction. The higher plaque binding observed for lecanemab compared with ACU193 may point to differences in plaque mobilization. In addition to binding characteristics, human IgG subclasses differ in structural and effector properties that have been proposed to influence ARIA risk. Human IgG1 and IgG2 antibodies differ in hinge region structure, complement activation, and Fcγ engagement. IgG1 antibodies more strongly activate complement and bind multiple Fcγ receptor types, which have been associated with antibody‐dependent cellular toxicity (ADCC), whereas IgG2 antibodies exhibit reduced complement activation and preferentially bind to FcγRII.^62^ These sub‐class‐specific differences in effector function have been suggested to contribute to differential inflammatory responses and may be relevant to ARIA risk observed with anti‐Aβ antibodies.

We next characterized cerebrovascular labeling. A previous qualitative study in fresh‐frozen human AD frontal cortex tissue described both lecanemab and ACU193 staining vascular Aβ deposits.^51^ In APP:hE4 mice, cortical vascular staining was low for both antibodies, consistent with the limited cortical CAA burden in this model. In the cerebellum, where vascular deposition was more robust, lecanemab exhibited significantly greater vascular labeling than ACU193 but did not reach pan‐Aβ or AMG levels. To better understand this difference, we examined the colocalization of the mAbs with AMG, a styrylbenzene derivative with similar staining patterns to Thioflavin S and Congo Red,^43^ which label CAA. Both mAbs showed a modest fraction of signal overlapping with AMG, suggesting labeling of vascular Aβ aggregates not detected by AMG and reflecting vascular Aβ heterogeneity. A larger fraction of AMG signal overlapped with lecanemab than ACU193, indicating that lecanemab labels a broader portion of β‐sheet‐ordered vascular Aβ, while also binding adjacent aggregates not recognized by AMG. Together, these results suggest that lecanemab binds Aβ species more closely associated with the vasculature than ACU193. Clinically, lecanemab has shown a lower incidence of ARIA in phase 3 trials relative to mAbs that bind more fibrillar species, including bapineuzumab, aducanumab, and donanemab,^49^, ^63^, ^64^, ^65^, ^66^ although titration to donanemab's target dose lowered ARIA rates.^67^ ACU193 is currently in a phase 2 clinical trial, and clinical ARIA rates are not yet available. Given that ARIA may be linked to antibody engagement with CAA,^24^, ^25^, ^26^, ^27^ these differences may influence relative ARIA risk. In *APOE* ε4 carriers, who typically exhibit greater CAA burden,^33^ increased antibody binding to vascular amyloid may occur. Further research is necessary to investigate whether the lower complement activation associated with human IgG2 mAbs could potentially reduce ARIA risk despite this increased CAA burden.

Several limitations should be considered. The small number of animals examined limited which comparisons could be evaluated using formal statistics. Sex‐specific effects were not analyzed due to limited sample size and were therefore not addressed in this study. As each mouse contributed a single tissue section per concentration, concentration‐specific comparisons were not subjected to inferential analyses, whereas pooled analyses of the same data supported statistical evaluation. Recombinant lecanemab was used for staining rather than the bona fide clinical antibody Leqembi. Comparisons between tissue fixed for 30 min versus 24 h reflect differences in fixation and tissue‐processing protocols, rather than fixation time alone. Although fixation duration was the primary variable of interest, associated processing steps may also contribute to observed differences in antibody labeling, and fixation‐based comparisons should be interpreted accordingly. A limitation of this study is that vascular amyloid labeling was classified using conservative, morphology‐based criteria as a vascular‐associated labeling pattern, rather than by definitive marker‑based confirmation of vascular origin in every instance. The approach prioritized unambiguous vascular‐associated labeling and may therefore under‐represent small‐diameter vessels or tangentially sectioned vascular amyloid. Accordingly, vascular labeling measurements should be interpreted as conservative, relative estimates rather than as an exhaustive detection of all vascular‑associated amyloid, and this approach was applied consistently across antibodies and conditions. Thin (10 µm) sections were selected to enable robust, side‐by‐side quantitative comparisons of antibody labeling across concentrations and antibodies within equivalent anatomical contexts. While thicker sections combined with z‐stack imaging and vascular marker double labeling may provide improved three‐dimensional resolution of vascular amyloid localization, such approaches would trade off quantitative comparability across mAb concentrations. Future studies using these complementary methods may further refine vascular and plaque localization of anti‑Aβ mAb labeling.

Aβ heterogeneity also limits interpretation. Aβ assemblies are polymorphic^68^ and transient,^69^ and a high degree of Aβ heterogeneity is observed in the human AD population.^70^, ^71^, ^72^ Any experimental measurement therefore represents a momentary snapshot of a dynamic population, which may not reflect in vivo conditions because this is an antibody applied to fixed brain sections, and antibody access to the brain in the clinic is routed from blood with only a fraction entering the brain. This might affect vascular preference and engagement as well as change competition dynamics when labeling and complicates extrapolation of our findings to human pathology. No mouse model fully recapitulates the complex pathology of AD. Aβ plaque morphology varies across APP transgenic lines,^73^, ^74^ and plaques in mouse models differ from human AD in structure, composition,^75^ and regional CAA distribution. In the APP:hE4 mouse model, CAA is predominantly located in the cerebellum with less in cortex. In humans with AD, CAA predominantly affects posterior brain regions,^76^, ^77^ while reports of cerebellar CAA are inconsistent, with most studies finding minimal or no involvement^78^, ^79^, ^80^ and others reporting relatively high deposition.^76^, ^81^ Further characterization of anti‐Aβ mAb binding on human AD tissue is under way to help bridge these gaps and support translation of our results.

In conclusion, these findings highlight how tissue preparation, including fixation, and mAb concentration influence Aβ immunolabeling. Lecanemab displayed greater plaque and vascular staining, consistent with recognition of a broader range of Aβ aggregates, whereas ACU193 appeared more selective in its staining profile. These results highlight differences in mAb binding preferences that could have implications for ARIA risk and plaque reduction in AD patients; however, future studies are needed to better understand the impact of other factors such as antibody effector function, titrated dosing, and route of administration on potential clinical outcomes.

## CONFLICT OF INTEREST STATEMENT

Martine B. Grenon declares no conflicts of interest. Erika N. Cline, Jasna Jerecic, Elizabeth Johnson, and Eric Siemers are employees and minor shareholders of Acumen Pharmaceuticals. Cynthia A. Lemere is a consultant and minor shareholder of Acumen Pharmaceuticals and consults or serves on scientific advisory boards for Advantage Therapeutics, Apellis Pharmaceuticals, Cyclo Therapeutics, Eli Lilly, Merck, MindImmune, Novo Nordisk, Receptive Bio, Switch Therapeutics, and Therini Bio. Author disclosures are available in the Supporting Information.

## CONSENT STATEMENT

This study did not involve human subjects, so informed consent was not required.

## Supporting information




**Supporting Information**: alz71509‐sup‐0001‐SuppMat.docx

**Supporting Information**: alz71509‐sup‐0002‐SuppMat.pdf
