# Supplementary material for: Ex vivo comparison of ACU193 and lecanemab reveals binding differences in mouse brain
Source: Alzheimers Dement. 2026 Jun 1;22(6):e71509. doi: 10.1002/alz.71509 (PMC13239446; doi:10.1002/alz.71509)
Supplement: Supplementary file 1 — Supporting Information: alz71509‐sup‐0001‐SuppMat.docx [file ALZ-22-e71509-s002.docx]

**Supplementary Table 1. Age, sex, and fixation conditions of APP:hE4 mice.**

| **Fixation Time**  **(in 4% PFA)** | **Age**  **(months)** | **Sex** |
| --- | --- | --- |
| 30 min | 21.2 | Female |
| 30 min | 20.0 | Male |
| 30 min | 20.0 | Male |
| 24 h | 19.6 | Female |
| 24 h | 19.6 | Male |
| 24 h | 20.0 | Male |

*PFA: Paraformaldehyde.

**
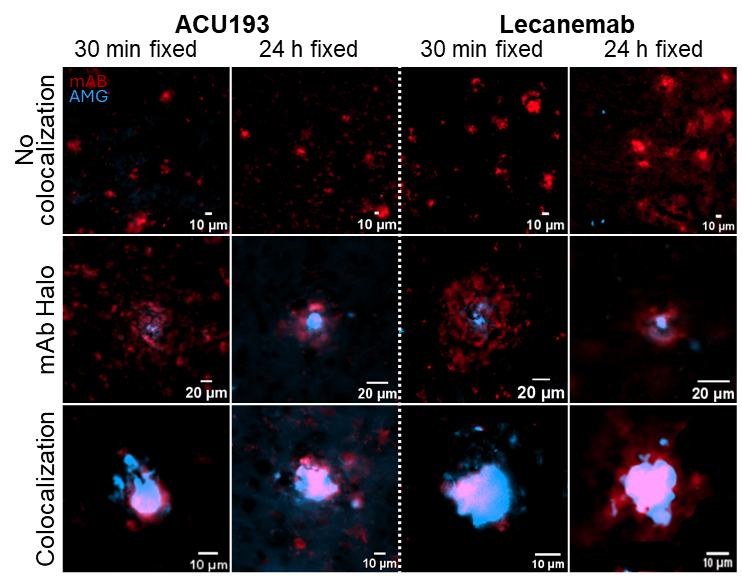
**

**Supplementary Fig. 1. Additional characterization of cortical plaque staining.** Representative merged microphotographs of serial sagittal sections fixed for 30 min or 24 h double labeled with 2.0 µg/ml of ACU193 or lecanemab (red), and AMG (blue). Examples are shown of mAb labeling with no AMG colocalization, mAb labeling surrounding AMG core and mAb colocalization with AMG. AMG: Amylo-Glo; mAb: Monoclonal antibody.

**
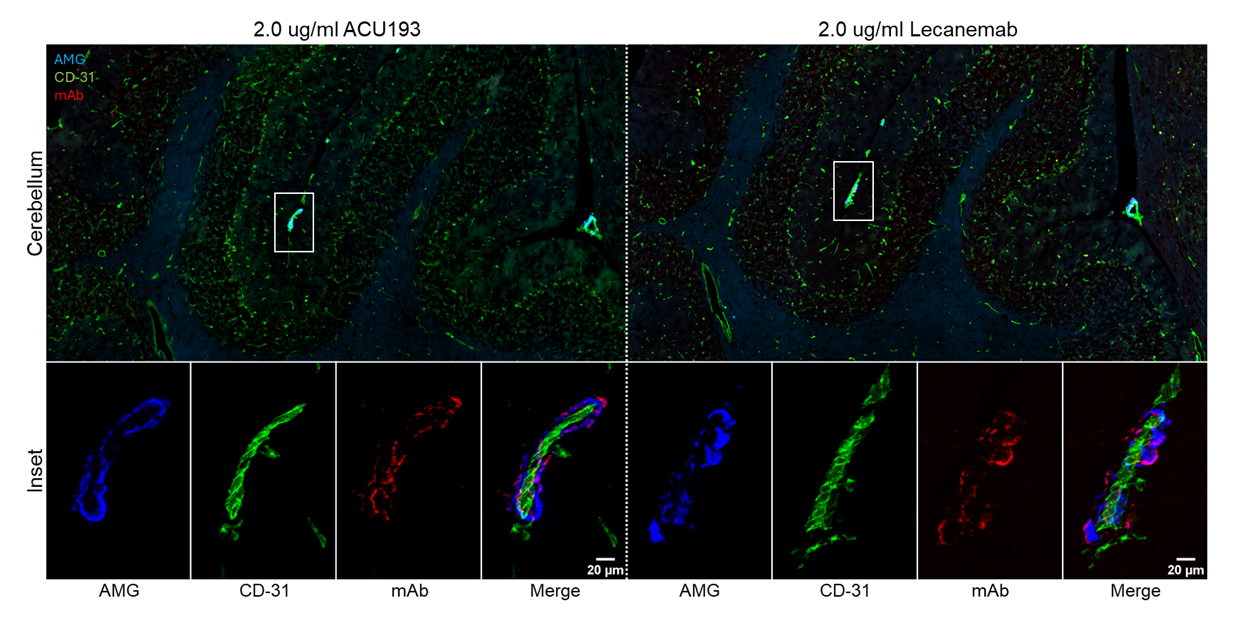
** **Supplementary Fig. 2. Verification of vascular staining.** Serial sagittal sections fixed for 24 h were triple labeled with 2.0 µg/ml of ACU193 or lecanemab, AMG and anti-CD-31, an endothelial cell marker. Microphotographs of cerebellar region. Merged images demonstrate mAb and AMG vascular-associated staining identified by colocalization with CD31-positive vasculature. These representative images validate our ability to identify vascular labeling based on morphology in subsequent analyses where a vascular marker was not used. AMG: Amylo-Glo; mAb: Monoclonal antibody.

**
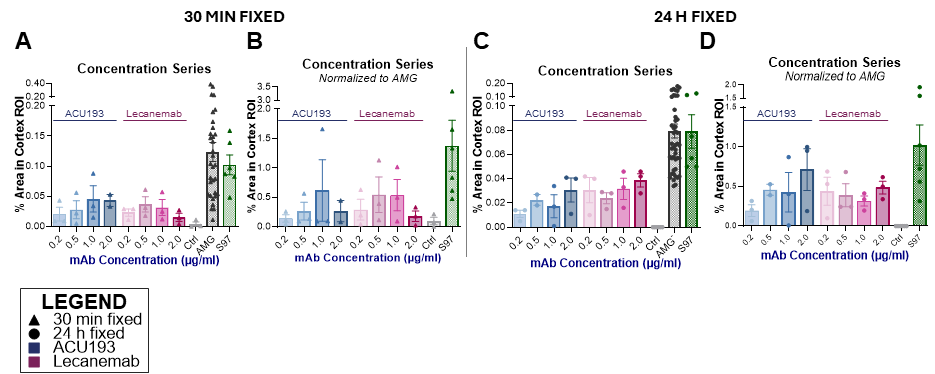
**

**Supplementary Fig. 3. Vascular immunoreactivity in the cortex; concentration effects.** Serial sagittal sections fixed for 30 min or 24 h were double labeled with a mAb (ACU193 or lecanemab; 0.2-2.0 µg/ml), and AMG. (A–D) Non-statistical quantification of vascular % area staining in the cortex in 30 min fixed tissue (A–B) and 24 h fixed tissue (C–D). Raw values are shown in (A, C); values in (B, D) are normalized to AMG of the same section to account for variability in amyloid load (graphs not statistically analyzed). All values are reported as mean or group means ± SEM. mAb: Monoclonal antibody; AMG: Amylo-Glo; Ctrl: Secondary antibody only control (anti-human IgG; no primary antibody).
